# Supplementary material for: The E3 ubiquitin ligase mechanism specifying targeted microRNA degradation
Source: Nature. 2026 Mar 18;652(8110):784–93. doi: 10.1038/s41586-026-10232-0 (PMC13083262; doi:10.1038/s41586-026-10232-0)
Supplement: Supplementary file 2 — Reporting Summary [file 41586_2026_10232_MOESM2_ESM.pdf]

Corresponding author(s): Brenda A. Schulman, David P. Bartel

Last updated by author(s): January 21, 2026

Reporting Summary

Nature Portfolio wishes to improve the reproducibility of the work that we publish. This form provides structure for consistency and transparency in reporting. For further information on Nature Portfolio policies, see our Editorial Policies and the Editorial Policy Checklist.

Statistics

For all statistical analyses, confirm that the following items are present in the figure legend, table legend, main text, or Methods section.

n/a	Confirmed	
☐	☒	The exact sample size (*n*) for each experimental group/condition, given as a discrete number and unit of measurement
☐	☒	A statement on whether measurements were taken from distinct samples or whether the same sample was measured repeatedly
☐	☒	The statistical test(s) used AND whether they are one- or two-sided<br>*Only common tests should be described solely by name; describe more complex techniques in the Methods section.*
☒	☐	A description of all covariates tested
☐	☒	A description of any assumptions or corrections, such as tests of normality and adjustment for multiple comparisons
☐	☒	A full description of the statistical parameters including central tendency (e.g. means) or other basic estimates (e.g. regression coefficient) AND variation (e.g. standard deviation) or associated estimates of uncertainty (e.g. confidence intervals)
☐	☒	For null hypothesis testing, the test statistic (e.g. *F*, *t*, *r*) with confidence intervals, effect sizes, degrees of freedom and *P* value noted<br>*Give P values as exact values whenever suitable.*
☒	☐	For Bayesian analysis, information on the choice of priors and Markov chain Monte Carlo settings
☒	☐	For hierarchical and complex designs, identification of the appropriate level for tests and full reporting of outcomes
☒	☐	Estimates of effect sizes (e.g. Cohen's *d*, Pearson's *r*), indicating how they were calculated

Our web collection on statistics for biologists contains articles on many of the points above.

Software and code

Policy information about availability of computer code

Data collection	Cryo-EM: SerialEM (v4.1); Flow cytometry: BD FACSDiva (v9.0); Gel and blot imaging: Amersham Typhoon, Typhoon 9410, and LI-COR Odyssey CLx; sRNA-seq: Illumina NovaSeq 6000; Bio-layer interferometry: Octet Data Acquisition HT (v13.0.1)
Data analysis	Cryo-EM structure analysis: CryoSparc (v6.4.2), COOT (0.9.8.95), Phenix (1.21.1), AlphaFold3, and ChimeraX (v1.8–1.9); Flow cytometry analysis: FlowJo (v10.10.0); Gel band quantification: ImageQuant TL (v10.2) and LI-COR ImageStudio (v6.1.0.79); Bio-layer interferometry analysis: Octet Data Analysis HT (v13.0.1); All statistical analyses: GraphPad Prism (v10.4.0); sRNA-seq analysis: cutadapt (v4.8), FASTX Toolkit (v0.0.14), and DESeq2 (v1.38.3). Original code for the analysis of sRNA-seq data is available publicly at https://github.com/lwblodgett/ZSWIM8_sensitivity_of_miRNA_isoforms.git (copy archived at Zenodo: https://doi.org/10.5281/zenodo.18265217).

For manuscripts utilizing custom algorithms or software that are central to the research but not yet described in published literature, software must be made available to editors and reviewers. We strongly encourage code deposition in a community repository (e.g. GitHub). See the Nature Portfolio guidelines for submitting code & software for further information.

Data

Policy information about availability of data

All manuscripts must include a data availability statement. This statement should provide the following information, where applicable:

- Accession codes, unique identifiers, or web links for publicly available datasets
- A description of any restrictions on data availability
- For clinical datasets or third party data, please ensure that the statement adheres to our policy

The structural data will be made publicly available from the PDB and EMDB upon manuscript publication. The atomic coordinates have been deposited in the PDB with accession code 9RWZ, and electron microscopy maps deposited with the Electron Microscopy Data Bank with codes EMD-54348, EMD-54349, EMD-54350, EMD-54351, and EMD-54352. Sequencing data has been deposited in the Gene Expression Omnibus with accession code GSE303177 and will be made publicly available upon manuscript publication. Uncropped in-gel fluorescence images, the workflow for cryo-EM structure generation, and gating strategies for flow cytometry experiments are provided in the Supplementary Figures.

Research involving human participants, their data, or biological material

Policy information about studies with human participants or human data. See also policy information about sex, gender (identity/presentation), and sexual orientation and race, ethnicity and racism.

Reporting on sex and gender	This study does not involve human participants, their data, or their biological material.
Reporting on race, ethnicity, or other socially relevant groupings	This study does not involve human participants, their data, or their biological material.
Population characteristics	This study does not involve human participants, their data, or their biological material.
Recruitment	This study does not involve human participants, their data, or their biological material.
Ethics oversight	This study does not involve human participants, their data, or their biological material.

Note that full information on the approval of the study protocol must also be provided in the manuscript.

Field-specific reporting

Please select the one below that is the best fit for your research. If you are not sure, read the appropriate sections before making your selection.

☒ Life sciences ☐ Behavioural & social sciences ☐ Ecological, evolutionary & environmental sciences

For a reference copy of the document with all sections, see nature.com/documents/nr-reporting-summary-flat.pdf

Life sciences study design

All studies must disclose on these points even when the disclosure is negative.

Sample size	No statistical methods were used to predetermine sample size. Sample sizes were chosen based on pilot experiments to ensure clear and reliable interpretation of the results. The sample sizes used in this study are consistent with standard practices in the field (PMID: 32661162, 33536622). The exact sample size is indicated in each figure legend.
Data exclusions	No data were excluded.
Replication	Experiments were replicated at least twice with similar results. Representative images are shown where appropriate.
Randomization	Not applicable; there was no subjective rating of data involved in our study.
Blinding	Not applicable; there was no subjective rating of data involved in our study.

Reporting for specific materials, systems and methods

We require information from authors about some types of materials, experimental systems and methods used in many studies. Here, indicate whether each material, system or method listed is relevant to your study. If you are not sure if a list item applies to your research, read the appropriate section before selecting a response.

Materials & experimental systems

n/a	Involved in the study
☐	☒ Antibodies
☐	☒ Eukaryotic cell lines
☒	☐ Palaeontology and archaeology
☒	☐ Animals and other organisms
☒	☐ Clinical data
☒	☐ Dual use research of concern
☒	☐ Plants

Methods

n/a	Involved in the study
☒	☐ ChIP-seq
☐	☒ Flow cytometry
☒	☐ MRI-based neuroimaging

Antibodies

Antibodies used

Primary antibodies: rabbit anti-ZSWIM8 (1:400; Invitrogen, PA5-59492), rabbit anti-HA (1:5,000; Cell Signaling Technology, C29F4, 3724), mouse anti-GAPDH (1:2,000; Invitrogen, GA1R, MA5-15738).
Secondary antibodies: IRDye 680RD goat anti-rabbit (1:10,000; LI-COR, 926-68071), IRDye 800CW goat anti-mouse (1:10,000, LI-COR, 926-32210).

Validation

anti-ZSWIM8: https://www.thermofisher.com/antibody/product/ZSWIM8-Antibody-Polyclonal/PA5-59492
anti-HA: https://www.cellsignal.com/products/primary-antibodies/ha-tag-c29f4-rabbit-mab/3724?srsltid=AfmBOopj1hBE5_slmYHbedpnQ-KkBtzFCpzgMNo4rj3eqg7Z7JtCB9YW
anti-GAPDH: https://www.thermofisher.com/antibody/product/GAPDH-Loading-Control-Antibody-clone-GA1R-Monoclonal/MA5-15738

Eukaryotic cell lines

Policy information about cell lines and Sex and Gender in Research

Cell line source(s)

Sf9 cells were were obtained from Thermo Fisher Scientific (11496015). High Five cells (BTI-TN-5B1-4) were obtained from Thermo Fisher Scientific (B85502). Expi293F cells were obtained from Thermo Fisher Scientific (A14527). K562 cells harboring a miR-7-sensitive GFP reporter were a gift from Joshua Mendell, and were originally obtained from American Type Culture Collection (ATCC). MEF, S2, and HEK293FT cells are Bartel lab stocks. Generation of specific cell lines was performed as described in the methods.

Authentication

Cell lines were not authenticated.

Mycoplasma contamination

Cell lines tested negative for mycoplasma contamination upon arrival to the lab.

Commonly misidentified lines
(See ICLAC register)

No commonly misidentified cell lines were used in this study.

Plants

Seed stocks

No plant material was used in this study.

Novel plant genotypes

No plant material was used in this study.

Authentication

No plant material was used in this study.

Flow Cytometry

Plots

Confirm that:

☒ The axis labels state the marker and fluorochrome used (e.g. CD4-FITC).

☒ The axis scales are clearly visible. Include numbers along axes only for bottom left plot of group (a 'group' is an analysis of identical markers).

☒ All plots are contour plots with outliers or pseudocolor plots.

☒ A numerical value for number of cells or percentage (with statistics) is provided.

Methodology

Sample preparation	Samples were prepared as described in the methods. Upon harvesting, cells were concentrated to 2 million cells/mL in media and subjected to flow cytometry.
Instrument	BD LSR Fortessa
Software	BD FACSDiva (v9.0); FlowJo (v10.10.0)
Cell population abundance	For each sample, 20,000 live cells were analyzed for GFP fluorescence in order to obtain a sufficient representation of each sample population.
Gating strategy	Initial gating steps included identification of live cells (SSC-A/FSC-A) followed by identification of single cells (FSC-H/FSC-A). This population of cells was analyzed by plotting the GFP fluorescence as a histogram. The gating strategy is shown in Supplementary Figure 7.

☒ Tick this box to confirm that a figure exemplifying the gating strategy is provided in the Supplementary Information.
