# Supplementary material for: The E3 ubiquitin ligase mechanism specifying targeted microRNA degradation
Source: Nature. 2026 Mar 18;652(8110):784–93. doi: 10.1038/s41586-026-10232-0 (PMC13083262; doi:10.1038/s41586-026-10232-0)
Supplement: Supplementary file 3 — Supplementary Tables 1–5. [file 41586_2026_10232_MOESM3_ESM.zip › 41586_2026_10232_MOESM3_ESM/2025-07-18813B-s3/Supplementary_Table_legends.docx]

**Supplementary Table 1** Table listing sequences of all target RNAs used in this study.

**Supplementary Table 2** Table describing all ZSWIM8 and AGO2 variants used in this study.

**Supplementary Table 3** Table listing sequences of all proteins used in this study.

**Supplementary Table 4** Table listing RNA, DNA, and LNA oligos used in this study.

**Supplementary Table 5** Tables with processed sRNA sequencing data.
